# Survival patterns of patients on maintenance hemodialysis for end stage renal disease in Ethiopia: summary of 91 cases

**DOI:** 10.1186/1471-2369-14-127

**Published:** 2013-06-19

**Authors:** Tamiru Shibiru, Esayas Kebede Gudina, Belete Habte, Amare Derbew, Tewodros Agonafer

**Affiliations:** 1 Jimma University, Jimma, Ethiopia; 2 St Gabriel General Hospital, Addis Ababa, Ethiopia

## Abstract

**Background:**

The increasing incidence and prevalence of chronic kidney disease is an important challenge for health systems around the world. Access for care of the disease in Ethiopia is extremely limited. The main purpose of the study was to investigate survival pattern and assess risk factors for poor outcome of patients on maintenance hemodialysis for end stage renal disease in Ethiopia.

**Methods:**

Medical records of patients on maintenance hemodialysis for end stage renal disease at Saint Gabriel General Hospital between 2002 and 2010 were reviewed. The data was collected by complete review of patient’s clinical data. Descriptive statistics was used for most variables and Chi-square test, where necessary, was used to test the association among various variables. Kaplan-Meier survival analysis was done to assess both short and long term survival. P-values of < 0.05 were considered as statistically significant.

**Results:**

A total of 190 patients were registered for hemodialysis at the hospital 91 of which were included in the final assessment. Mean age at dialysis initiation was 58 ± 15 years. Fifty-five (60.4%) of the patients had prior history of diabetes. Almost all of them had serum creatinine of > 5mg/dl and some degree of anemia at dialysis initiation. Forty-one (45.1%) deaths occurred during dialysis treatment and 21 (23.1%) of patients died within the first 90 days of starting dialysis. Only 42.1% of them survived longer than a year. The frequently registered causes of death were septicemia (34.1%) and cardiovascular diseases (29.3%). Use of catheter as vascular access was associated with decreased short term and long term survival.

**Conclusion:**

Dialysis as treatment modality is extremely scarce in Ethiopia and affordable to only the rich. Survival pattern in those on the treatment is less satisfactory and short of usual standards in the developed world and needs further investigation. We thus recommend a large scale analysis of national dialysis registry at all dialysis centers in the country.

## Background

Chronic kidney disease (CKD) is an important challenge for health systems around the world [1] consuming a huge proportion of health care finances [2,3]. It is even more significant for developing countries [1,4] which now face the double burden of infectious diseases and growing problems of non-communicable diseases such as obesity, diabetes and hypertension [5]. About 85% of the world populations live in less developed part of the world where CKD prevention programs are either rudimentary or virtually nonexistent [3]. Morbidities and mortalities emanating from CKD in these countries are immense and related to limited access for treatment options [2].

Renal replacement therapy (RRT) is the mainstay of care for patients with end stage renal disease (ESRD). Dialysis as an option of RRT prolongs survival, reduces morbidities and improves quality of life. However, despite many technical advances, morbidities and mortalities of patients on dialysis remain unacceptably high and their quality of life is often poor [6]. Common independent predictors of survival are age, race, serum albumin at the start of dialysis, activity level at the start of dialysis, and presence of certain comorbidities such as heart failure and cancer [7].

Treatment options for CKD are not readily available for most countries in sub-Saharan Africa. The region contributes to less than 5% of patients on RRT worldwide [5,8-10]. Dialysis and transplant programs in this part of the world are dependent on the availability of external funding and donors. As a result, only less than 5% of patients with diagnosed ESRD are able to get treatment for longer than 3 months [2].

For many years the magnitude of ESRD in Ethiopia has not been studied. The use of dialysis in the country as a treatment strategy for ESRD dates less than a decade. In addition, access for dialysis is limited and is a highly unaffordable for the general public. Each dialysis session costs about $100 (1700 Birr) excluding the costs for other supportive cares. Because of the low socio-economic status, dialysis is thus considered as luxury care in the country. There is currently no dialysis center in Public hospitals in Ethiopia with a population surpassing 85 million. In addition, there is no national strategy for prevention and care of patients with CKD.

The aim of this study was to investigate survival patterns of patients on maintenance hemodialysis for end stage renal disease and assess factors related to poor outcomes.

## Methods

### Setting

This is a retrospective analysis of patients’ clinical data on maintenance hemodialysis for ESRD at St Gabriel General Hospital between February 2002 and August 2010. The hospital is located in Addis Ababa, Ethiopia. It is the first private Hospital established in 1995 and one of the three dialysis centers in the country. By the time the study was conducted, the center had three dialysis machines operated by two dialysis nurses, a general practitioner with short term training on dialysis and one nephrologist.

### Participants

Patients on maintenance hemodialysis for ESRD at the hospital between February 2002 and August 2010.

### Inclusion criteria

All adult patients (18 years and older) who were on maintenance hemodialysis for ESRD during the specified period were included in the study.

### Exclusion criteria

1. Patients who started on hemodialysis for acute renal failure.

2. Incomplete medical records

3. Patients on transient hemodialysis (patients from abroad who took maintenance dialysis at the Centre for short period of time)

### Selection of participants

Dialysis registration book was reviewed to identify patients who were eligible for the study. The charts of all eligible patients were then identified for review.

### Data collection

The data was collected by using pretested structured questionnaire that consisted of characteristics related to demographic profiles, causes and risk factors of CKD, clinical conditions of patients at initiation and last session of dialysis and treatments given. These were collected by reviewing patients’ medical records and dialysis registration book.

### Data quality control

Data collectors were trained and supervised during data collection. Charts were coded to avoid repetition of recording and findings were cross-checked with dialysis registration book. The collected data were also checked for completeness and internal consistency.

### Data processing, analysis and interpretation

The data were coded, cleaned, entered and analyzed with the help of SPSS version 16. Multivariate analysis and other descriptive statistics were used for most variables and Chi-square test, where necessary, was used to test the association among various variables. Kaplan- Meier survival analysis was used to assess the time to death after initiation of dialysis treatment. P-values of < 0.05 were considered as statistically significant.

### Operational definitions

Patient outcome in this study refers to condition at the final session of dialysis – dead or alive. Only those deaths without any other plausible explanations (in the absence of any other immediate condition independent of ESRD like trauma) were recorded as relevant. Deaths in the first ninety days of initiation of dialysis were regarded as early mortality.

### Ethical considerations

Ethical approval was obtained from Jimma University Ethical review board. The information obtained from the chart will remain confidential indefinitely.

## Result

A total of 190 patients were registered for hemodialysis at the hospital between 2002 and 2010. Only 91 patients were included for final outcome analysis; the rest were excluded for different reasons.

### Baseline characteristics at initiation of dialysis

Table 1 summarizes background characteristics of 91 patients that were included in the study. The mean age at dialysis initiation was 58 ± 15 years (range 19 – 86 years). Only 5.5% of them were from outside Addis Ababa (Table 1).

**Table 1:** Background characteristics of 91 patients on maintenance hemodialysis for end stage renal disease in Ethiopia

**Characteristics**	**No (%)**
**Sex**	
Male	56(61.5)
Female	35(38.5)
**Age**	
19-34	10(11.0)
35-64	43(47.2)
≥65	38(41.8)
**Address of origin**	
Addis Ababa	86(94.5)
Other	5(5.5)
**Potential causes of CKD identified**	
Diabetes mellitus	50(54.9)
Hypertension	18(19.8)
Glomerulonephritis	16(17.6)
Diabetes mellitus and hypertension	5(5.5)
Polycystic kidney disease	1(1.1)
Multiple myeloma	1(1.1)
**Vascular access**	
Fistula	41(45.1)
Catheter	38(41.8)
Graft	12(13.2)

*CKD* Chronic Kidney disease.

Fifty-five (60.4%) of the patients had prior history of diabetes, 5 of which had both diabetes and hypertension (Table 1).

The mean serum creatinine at dialysis initiation was 10.7 ± 3.4.mg/dl. All but one patient had creatinine > 5mg/dl. Over half of them had creatinine > 10mg/dl. Virtually all of them had some degree of anemia at initiation of dialysis. The mean hematocrit was 29.5 ± 4.5% (range 18.8% to 40%).

Arteriovenous fistula and catheter were the predominant vascular accesses used for hemodialysis at the hospital (Table 1).

### Treatment modalities

Most of the patients (87.9%) had at least two sessions of dialysis a week. The duration of each session ranged between 3 to 4 hours (Table 2). On average, patients took maintenance hemodialysis for a median of 132 days. Two patients took only one session of dialysis and died immediately. Only one patient survived longer than 6 years taking a total of 742 dialysis sessions.

**Table 2:** Treatment modalities for 91 patients on maintenance hemodialysis for end stage renal disease in Ethiopia

**Characteristics**	**N (%)**
Frequency of dialysis sessions/week	
Once	11(12.1)
Twice	51(56.0)
Three times	29 (31.9)
Duration of dialysis per session	
3 hours	3(3.3)
3^1/2^ hours	84(92.3)
4 hours	4(4.4)
EPO treatment	
Yes	53(58.3)
No	38(41.8)
Blood transfusion	
Yes	59(64.8)
No	32(35.2)

*EPO* Erythropoietin.

About 74% of patients were given some forms of medications for complications of CKD, 58.3% were given erythropoietin, 43% of which were given regularly. Fifty-nine (64.8%) of them have been given blood transfusion at least once during their time on dialysis (Table 2).

### Comorbidities

Some forms of comorbid conditions occurred in 47 (51.6%) of the patients. The most common comorbidity identified was cardiovascular disease (CVD) which was recorded in 29.7% of the cases. Five of CVD cases were acute myocardial infarction. Thirteen (14.3%) of them developed sepsis sometime during their course. Intradialytic hypotension occurred in 38.5% of patients at least once during the period on dialysis.

### Outcome assessment

The mean creatinine and hematocrit levels at the last session of dialysis were 8.7 ± 3.1mg/dl and 29.8 ± 5% respectively. A paired t-test showed that serum creatinine at the last session of dialysis decreased by 1.97mg/dl from the value at initiation of dialysis (p < 0.0001). The blood urea nitrogen (BUN) also significantly dropped from pre-dialysis level of 118.9 ± 45.0mg/dl to 92.8 ± 47.5mg/dl at the last session (p < 0.0001). However, there was no significant change in hematocrit level.

Forty-one (45%) deaths occurred during dialysis treatment; 21 of the deaths occurred within the first 90 days of starting dialysis. Thirty-six deaths occurred at the hospital and the remaining 5 occurred at home. Survival at three month and one year was 61.5% and 42.1% respectively. The frequently registered causes of death were septicemia (34.1%) followed by cardiovascular diseases (29.3%) (Table 3)

**Table 3:** Outcome of 91 patients on maintenance hemodialysis for end stage renal disease in Ethiopia

**Characteristics**	**N (%)**	**Male, N (%)**	**Female, N (%)**	**P – value**
**Outcome of patient**				0.915
Died	41(45.0)	26 (46.4)	15 (42.9)	
Alive	14(15.4)	8 (14.3)	6 (17.7)	
Unknown (left the center^‡^)	36 (39.6)	22 (39.3)	14 (40.0)	
**Causes of death**				
Sepsis	14(34.1)	8 (30.8)	6 (40.0)	0.886
Cardiovascular diseases	12(29.3)	8 (30.8)	4 (26.7)	
Uremic complications	10(24.4)	6 (23.1)	4 (26.7)	
Sudden death	1(2.4)	1 (3.8)	0	
Unknown	4(9.6)	3 (11.5)	1 (6.7)	

^**‡**^10 patients discontinued dialysis, 9 referred for renal transplant abroad, 10 left the country, 7 transferred to other centers in the country.

There was no significant mortality difference between age groups, sex, serum creatinine and hematocrit levels at dialysis initiation. However, mortality markedly varied among the type of vascular access used with the highest observed among those using catheter (p < 0.0001).

**Figure 1:**
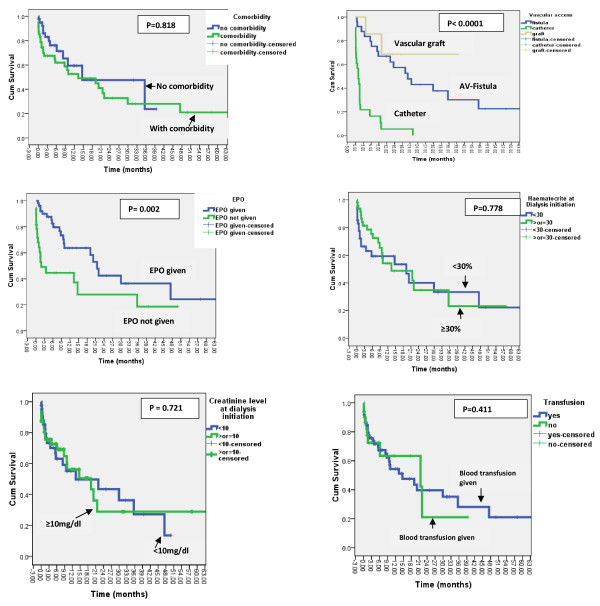
Kaplan – Meier survival curves in patients on dialysis in Ethiopia.

Kaplan – Meier survival analysis also showed that only the type of vascular access used and erythropoietin treatment significantly affected both short-term and long term survival patterns. The median survival in patient using catheter, fistula and vascular graft was 2.8, 27.2 and 39 months respectively (P < 0.0001). No patient using catheter survived longer than 21 months. Similarly, twelve month survival in patients who were given EPO was 53.2% as compared to 26.1% in those who were never given it (P = 0.002) (Figure 1).

The age and sex adjusted HR for catheter use was 8.399 (95% CI, 1.948 to 36.216; p = 0.004. For erythropoietin treatment, this was found to be 0.320 (95% CI, 0.165 to 0.623; p = .001).

## Discussion

Renal replacement therapy is an extremely scare treatment modality in Ethiopia and accessible to only few wealthy urban dwellers. Long term survival in those undergoing dialysis is very low and can be attributable to various medical conditions. Findings in this study are from one dialysis center in Ethiopia and can be used as a preliminary data for future studies and help improve care of such patients.

Most of the patients (60.4%) with ESRD at the hospital had prior diagnosis of diabetes mellitus. Even though this does not guarantee diabetes as the culprit, it is of paramount clinical importance as far as the disease burden is concerned. Only 17.6% of them were diagnosed with glomerulonephritis unlike other studies in Africa where it was found to be the commonest cause of CKD [8,11]. As there was no routine renal biopsy and other necessary investigations for diagnosis of renal diseases, the real causes of ESRD in this setting cannot be easily stated. In addition, findings from this study can never be used as a representative data for the country as most of the participants were from Addis Ababa where the living standard is better and hygiene is good.

Diagnosis of CKD was based on clinical ground only like elsewhere in Africa [11]. GFR was not done for most of the patients at initiation of dialysis and the criteria to start dialysis were based mainly on patients’ clinical conditions such as uremic symptoms and fluid overload intractable to medical treatments. However, the mean serum creatinine of 10.7mg/dl at initiation of dialysis was comparable with findings from different countries around the world [12,13].

The median survival was 263 days with 62.1% of patients surviving their 90^th^ day after starting dialysis. This figure is better than the finding in Ghanaian study with median survival of 90 days and only 45% of patients reaching their 90^th^ day after starting dialysis [11]. However, only 42.1% of the patients lived longer than one year after dialysis initiation. The 5 year survival rate was 14.8%. When compared with the long term survival in developed countries where the 2-, 5- and 10-year survival was 67, 35 and 11% respectively [14], this finding is negligible. These results pose a question on the adequacy of dialysis delivery in the country. However, other factors should be also considered and be addressed in future prospective studies.

Multivariate analysis did not show the influence of socio-demographic factors and supportive cares given on short-term and long survival. This can be explained by the fact that survival was very low among all groups. In addition, the small sample size in each group was not enough to detect the difference.

Septicemia accounted for 34.1% of all the causes of mortality. These were patients who had catheter as permanent vascular access, which may be explained by the presence of septicemia from potential catheter exit site. The most powerful predictor of patient survival was thus type of vascular access (p < 0.0001). Catheter as a vascular access was associated with poor outcome as compared to graft in Dialysis Outcomes and Practice Patterns Study (DOPPS) [15]. Similarly, in this study, 42% of patients had catheter as vascular access and the death toll in these groups was 96%, much higher than the other groups. Survival at 1 year was 5.4% for catheter group and 66.9% in other types of vascular accesses (p < 0.0001).

Although important findings were obtained from the present study, there are some limitations worth mentioning. The study was based on chart revision where incomplete documentation, inappropriate chart labeling and lost records made it difficult to include all patients registered for dialysis. Urea reduction ratio is the cheapest but an invaluable parameter to assess dialysis adequacy, however, it was inconsistently done and could not be used in this study. Complete anthropometric measurements, glomerular filtration rate, serum albumin and electrolyte were rarely done and could not be used for this study. As a result, it was difficult to state the overall clinical condition of patients at initiation of dialysis and causes of death as well. Furthermore, data was collected from one health care center, and thus a small sample size. Most of the patients were from the capital Addis Ababa; a region characterized by a better socio-economic status, and therefore, cannot be used as representative national data.

## Conclusion

Dialysis as treatment modality is extremely scarce in Ethiopia and affordable to only the rich. Its effect on improving survival is far short of the usual standards in the developed world and needs further investigation. We thus recommend a large scale analysis of national dialysis registry at all dialysis centers in the country. Furthermore, we urge all concerned to investigate the epidemiology of CKD in the country to plan further intervention.

## Competing interests

The authors declare that they have no competing interests.

## Authors’ contributions

TS designed the study, developed instruments, supervised data collection and data entry, participated in data analysis and manuscript writing. EKG analyzed the data and wrote the manuscript. BH and AD participated in the study design, supervised instrument development and contributed to manuscript editing. TA participated in instrument design and supervised data collection. All authors have read and approved the final manuscript.

## Pre-publication history

The pre-publication history for this paper can be accessed here:

http://www.biomedcentral.com/1471-2369/14/127/prepub
